# Identification of Multiple Dehalogenase Genes Involved in Tetrachloroethene-to-Ethene Dechlorination in a* Dehalococcoides*-Dominated Enrichment Culture

**DOI:** 10.1155/2017/9191086

**Published:** 2017-08-15

**Authors:** Mohamed Ismaeil, Naoko Yoshida, Arata Katayama

**Affiliations:** ^1^Department of Environmental Engineering and Architecture, Graduate School of Environmental Studies, Nagoya University, Chikusa, Nagoya 464-8601, Japan; ^2^Department of Civil Engineering, Nagoya Institute of Technology, Showa, Nagoya 466-8555, Japan; ^3^Department of Civil Engineering, Graduate School of Engineering, Nagoya University, Chikusa, Nagoya 464-8603, Japan; ^4^Institute of Materials and Systems for Sustainability (IMaSS), Nagoya University, Chikusa, Nagoya 464-8601, Japan

## Abstract

Chloroethenes (CEs) are widespread groundwater toxicants that are reductively dechlorinated to nontoxic ethene (ETH) by members of* Dehalococcoides*. This study established a* Dehalococcoides*-dominated enrichment culture (designated “YN3”) that dechlorinates tetrachloroethene (PCE) to ETH with high dechlorination activity, that is, complete dechlorination of 800 *μ*M PCE to ETH within 14 days in the presence of* Dehalococcoides* species at 5.7 ± 1.9 × 10^7^ copies of 16S rRNA gene/mL. The metagenome of YN3 harbored 18* rdhA* genes (designated* YN3rdhA1–18*) encoding the catalytic subunit of reductive dehalogenase (RdhA), four of which were suggested to be involved in PCE-to-ETH dechlorination based on significant increases in their transcription in response to CE addition. The predicted proteins for two of these four genes, YN3RdhA8 and YN3RdhA16, showed 94% and 97% of amino acid similarity with PceA and VcrA, which are well known to dechlorinate PCE to trichloroethene (TCE) and TCE to ETH, respectively. The other two* rdhAs*,* YN3rdhA6* and* YN3rdhA12,* which were never proved as* rdhA* for CEs, showed particularly high transcription upon addition of vinyl chloride (VC), with 75 ± 38 and 16 ± 8.6 mRNA copies per gene, respectively, suggesting their possible functions as novel VC-reductive dehalogenases. Moreover, metagenome data indicated the presence of three coexisting bacterial species, including novel species of the genus* Bacteroides*, which might promote CE dechlorination by* Dehalococcoides.*

## 1. Introduction

Chloroethenes (CEs) such as tetrachloroethene (PCE) and trichloroethene (TCE) have been used extensively in dry cleaning and as degreasing agents. Consequently, they are commonly detected in groundwater because of improper disposal and accidental spills. Dehalorespiring bacteria, such as* Dehalococcoides*, which reductively dechlorinate CEs via respiration [1], have been applied as biocatalysts to remedy environments contaminated with CEs [2]. However, the accumulation of toxic compounds such as* cis*-dichloroethene (*cis*-DCE) and vinyl chloride (VC) as incomplete dechlorination products due to the limited distribution of* Dehalococcoides*, which completely dechlorinate CEs to ethene (ETH), has been reported [2]. To date, only members of the genus* Dehalococcoides* are known to convert these toxic intermediates to nontoxic ETH [2]. So far, no isolated strain can completely dechlorinate PCE to ETH without accumulation of toxic intermediates in pure culture [3]. Complete dechlorination of PCE to ETH has been rarely demonstrated, even in complexed microbial communities, and the time required for PCE-to-ETH dechlorination in consortia is highly variable, depending on the microbial community and PCE concentration [4–7]. In general, consortia containing non-dehalorespiring bacteria together with* Dehalococcoides* show faster dechlorination [8, 9], and several possible functions of non-dehalorespirators in dehalorespiring consortia have been suggested [8–12].

The ability of* Dehalococcoides* to partially or completely dechlorinate CEs as well as other organohalides depends largely on reductive dehalogenase (Rdh). Rdhs identified in* Dehalococcoides* and other dehalorespiring bacteria consist of a catalytic subunit (RdhA) encoded by the* rdhA* gene and membrane-anchoring protein (RdhB) encoded by* rdhB* neighboring to* rdhA *[13, 14]. In addition, several other genes assumed as Rdh-associated genes are frequently located close to* rdhA* and* rdhB* and are suggested to be involved in transcription regulation (i.e.,* rdhS, rdhP*, and* rdhR*), transcription regulation/electron transport* (rdhC)*, or maturation of Rdhs (i.e.,* rdhF, rdhG*,* rdhH*, and* rdhI*) [1].

To date, several hundred* rdhA*s have been sequenced, although specific substrates have been determined for only a few RdhAs that were successfully partially purified from* Dehalococcoides*; VcrA [15] and BvcA [16] show apparent dechlorination of TCE to ETH and* cis-*DCE to ETH, respectively, while PceA [17] and TceA [18] can dechlorinate PCE to TCE and TCE to VC, respectively. Besides the CEs-RdhAs that were identified as RdhA for CEs on the basis of enzymatic activity, several RdhAs have been suggested to dechlorinate CEs on the basis of their high sequence similarity to these experimentally proven CE-RdhAs [19–21] and high transcription of* rdhA* specifically induced by CEs [22, 23]. Reverse transcription quantitative PCR (RT-qPCR) is a relatively straightforward and promising screening tool for finding novel functions of* rdhA* from the clear correlation between high transcription levels of known CE-RdhAs and their actual function [23, 24].

In this study, we successfully obtained a consortium containing* Dehalococcoides *that showed high, complete PCE-to-ETH dechlorination activity, that is, dechlorination of 800 *μ*M PCE to ETH within 14 days. Four* rdhA*s are suggested to be involved in PCE-to-ETH dechlorination on the basis of their transcription response to CEs. Notably, two out of the four* rdhA*s had been never proven as* rdhA* for CEs and, thus, are novel candidate* rdhA*s involved in CE dechlorination. In addition, metagenome data showed the presence of three coexisting bacterial species, including novel species of the genus* Bacteroides*, and suggested they might promote CE dechlorination by* Dehalococcoides*.

## 2. Materials and Methods

### 2.1. Enrichment of Bacteria Dechlorinating CEs

Sediment from Arako River (Nagoya city, Aichi prefecture, Japan) was used as the inoculum for the enrichment of CE-dechlorinating bacteria. Approximately 10 g (wet weight) of the sediment was introduced into 60 mL serum bottles containing 20 mL of distilled water supplemented with 1 mg/L resazurin. The bottles were purged with N_2_ gas for 15 min and sealed with Teflon-lined rubber stoppers and aluminum crimp caps. The bottles were purged once more with a gas mixture of H_2_ and CO_2_ (4 : 1, v/v) and then supplemented with 20 mM acetate and either PCE (1 mM) or* cis*-DCE (500 *μ*M) as an e^−^ acceptor and incubated at 28°C. After incubation for 1 month, 100 *μ*L of the headspace gas was analyzed in a gas chromatograph (GC) as described below.

Microcosms showing dechlorination of PCE or* cis*-DCE were transferred to 20 mL of a mineral medium (DHB-CO_3_ medium) in a 60 mL serum bottle at a 5% transfer rate. The DHB-CO_3_ medium included the following components (per liter): 1 g NaCl; 2.5 g NaHCO_3_; 0.5 g KCl; 0.5 g NH_4_Cl; 0.1 g CaCl_2_·2H_2_O; 0.1 g MgCl_2_·6H_2_O; 0.2 g KH_2_PO_4_; 1 mL of 1 mg/L resazurin solution; 1 mL of trace element solution SL10 [25]; 10 mL of vitamin solution [26]; 1 mL of Se/W solution [25]; and 10 mL of titanium (III) trinitriloacetic acid solution [27]. The medium was prepared under anaerobic condition with flashing of a mixture of N_2_ and CO_2_ (4 : 1, v/v), as described previously [28]. Prior to inoculation, the headspace was exchanged aseptically with a gas mixture of H_2_ and CO_2_ (4 : 1, v/v). The transferred cultures were repeatedly incubated and transferred every month after observation of the dechlorination. The cultures supplemented with PCE showed weak dechlorination activity, and therefore, further enrichment was stopped after the third transfer. On the other hand, cultures supplemented with* cis*-DCE showed stable and complete dechlorination activity. Therefore, only the culture supplemented with* cis*-DCE was maintained and was designated “YN3.”

Time-course monitoring of the CEs and dechlorinated products in YN3 was carried out in freshly prepared culture (20 mL) inoculated with YN3 from the previous passage (1 mL) and supplemented with either 70 *μ*M PCE or 500 *μ*M* cis*-DCE in 60 mL serum bottles. To assay the applicability of YN3 for dechlorination of high concentrations of PCE, YN3 grown with 70 *μ*M PCE was additionally spiked with 200–800 *μ*M PCE. A portion of YN3 was periodically withdrawn for monitoring of 16S rRNA gene copy number for total bacteria and specifically, for* Dehalococcoides*, as described below. To test the ability of YN3 to dechlorinate TCE,* trans*-DCE, or 1,1-DCE, 200 *μ*M of these compounds was added to cultures.

### 2.2. Chemical Analysis

CEs and ETH were detected and quantified by gas chromatography (GC), as described previously [36]. In brief, 100 *μ*L of headspace gas was withdrawn using a gastight syringe and injected manually into a GC-2014 (Shimadzu, Kyoto, Japan) equipped with flame ionization detector and Porapak Q column (GL Sciences Tokyo, Japan). Nitrogen was used as a carrier gas at a flow rate of 35 mL/min. The injection, detection, and column temperatures were set at 200°C.

### 2.3. DNA Extraction

For genomic DNA extraction, cells from YN3 were collected by centrifugation at 15,000 ×g for 15 min and stored at −20°C until DNA extraction. Genomic DNA was extracted as described previously [37].

### 2.4. Metagenome Sequencing, Sequence Analysis, and Annotation

Extracted DNA was prepared for sequencing by adding Illumina adaptor sequences, using the TruSeq DNA Sample Prep Kit (Illumina). Metagenome sequencing was carried out using a pair-end run on the Illumina HiSeq platform at Hokkaido System Science Co., Ltd. (Sapporo, Japan). Cutadapt (ver. 1.1 [38]) was used for trimming of the Illumina adaptor sequences and Velvet software (ver. 1.2.08 [39]) was used for sequence assembly. Gene prediction and annotation were carried out using the Microbial Genome Annotation (MiGAP) (ver. 2.19; http://www.migap.org) [40] and Rapid Annotation using Subsystem Technology (RAST) (ver. 2.0; http://rast.nmpdr.org) pipelines [41]. The contigs obtained from the metagenomic reads were taxonomically classified based on DNA sequence similarity of the entire contigs by NCBI BLASTN (https://blast.ncbi.nlm.nih.gov). In this study, contigs having at least 90% sequence similarity to a known bacterial sequence were classified at the genus level [42, 43]. Contigs showing poor similarity with partial sequences in the known genome were classified based on similarity of the translated sequences of individual genes presenting in the contigs with genes in the published genome by BLASTP. Predicted coding sequences (CDSs) of all contigs were classified into functional categories by matching them to the SEED database provided within the RAST pipeline [44]. The phylogenetic trees of 16S rRNA genes and RdhAs were constructed using MEGA 6 software [45].

### 2.5. Quantitative PCR (qPCR)

qPCR targeting 16S rRNA genes of* Dehalococcoides* and the total bacterial population was carried out using the primer sets shown in Table S1 (in Supplementary Material available online at https://doi.org/10.1155/2017/9191086) and the FastStart Essential DNA Green Master kit (Roche Diagnostics) on a LightCycler system (Roche Diagnostics, Mannheim, Germany), as described previously [46]. The primers were designed using the GenScript Real-Time PCR (TaqMan) Primer Design Tool (https://www.genscript.com/tools/real-time-pcr-tagman-primer-design-tool).

### 2.6. Reverse Transcription- (RT-) qPCR

mRNA copy numbers of transcribed* rdhA*s were examined by RT-qPCR in YN3 spiked with CEs, using specific primers (Table S1, supplementary material). The YN3 used for the RT-qPCR experiment was incubated for approximately 30 days to complete the dechlorination of PCE or* cis*-DCE to ETH, and the headspace gas was exchanged with filter-sterilized N_2_ to eliminate remaining CEs and ETH. It was then incubated for another 5 days without addition of any CEs as starvation period to attenuate mRNA in the cells. Twenty-milliliter aliquots of starved YN3 were transferred into serum bottles and spiked with PCE (100 *μ*M), TCE (200 *μ*M),* cis*-DCE (500 *μ*M), or VC (100 *μ*M). An unspiked culture was prepared in parallel as a negative control. After 9–15 h of culture showing dechlorination activity, cells were collected by centrifugation for RNA and DNA extractions. Cell pellets used for RNA extraction were spiked with mRNA of the luciferase-encoding gene (*luc*) (Promega, Fitchburg, WI, USA) and then subjected to RNA extraction using ISOGEN II (Nippon Gene, Tokyo, Japan). RT-qPCRs for* rdhA *and* luc* quantification were carried out in parallel using the One Step SYBR PrimeScript RT-PCR Kit II (TaKaRa, Otsu, Japan). mRNA copies of* rdhA* in YN3 were normalized to spiked-in* luc* mRNA. The RNA recovery rates of the samples in this study varied with total RNA concentration and were in the range of 2–17%. The transcription rate of* rdhA* was calculated by dividing the mRNA copy numbers by the gene copy numbers determined by qPCR in parallel.

## 3. Results

### 3.1. Enrichment of PCE-to-ETH Dechlorinating Culture

A serial transfer culture established from CE-contaminated river sediment, using 1 mM PCE and 500 *μ*M* cis*-DCE, yielded two enrichment cultures: an unstable PCE-dechlorinating culture and the stable* cis*-DCE-to-ETH-dechlorinating YN3 (Figure S1, supplementary material). YN3 maintained activity to dechlorinate 500 *μ*M* cis*-DCE to ETH within 10 days even after 50 transfers over five years. In contrast, the PCE-dechlorinating culture lost its dechlorination activity after the third transfer. Next, YN3 was tested for dechlorination of PCE at a low concentration (70 *μ*M) and showed stable PCE-to-ETH dechlorination activity.

### 3.2. PCE-to-ETH Dechlorination in YN3

**Figure 1:**
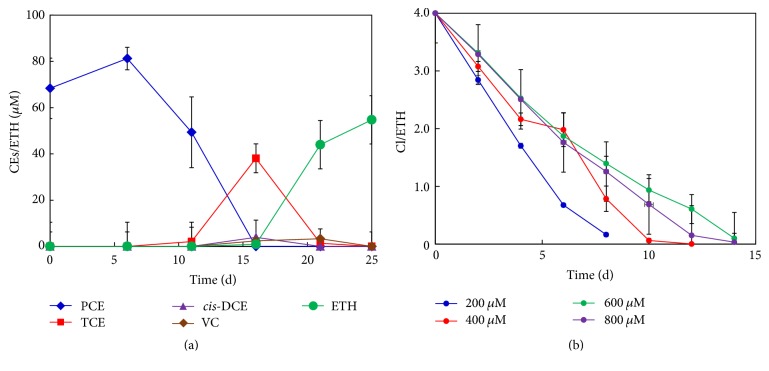
Reductive dechlorination of PCE to ETH by YN3. (a) Dechlorination of 70 *μ*M PCE in YN3. Error bars represent standard deviations (SDs, *n* = 6), (b) dechlorination of spiked PCE (200–800 *μ*M) to ETH in YN3 grown with 70 *μ*M PCE. The *y*-axis in panel (b) presents the average number of chlorines on ethylene. Error bars represent SDs (*n* = 3).

Figure 1(a) shows the changes in PCE and the dechlorination products in YN3 throughout incubation. The culture supplemented with 70 *μ*M PCE started to dechlorinate PCE to TCE at 6 days, reaching a maximum of 38 ± 6 *μ*M TCE at day 16. The dechlorination of TCE started at day 11, and the main dechlorination products of TCE were ETH together with* cis*-DCE and VC, indicating the immediate dechlorination of TCE to ETH via* cis*-DCE and VC in YN3. At day 25, the concentration of ETH reached a maximum (54 ± 2 *μ*M) and accounted for 77% of the added PCE. The results clearly demonstrated the ability of YN3 to completely dechlorinate PCE into ETH via TCE,* cis*-DCE, and VC. A PCE spiking experiment showed that YN3 could dechlorinate 200–800 *μ*M of PCE within 14 days (Figure 1(b)). YN3 also dechlorinated 200 *μ*M 1,1-DCE to ETH via VC within 2 weeks without the accumulation of intermediates; however, it showed no dechlorination activity for* trans*-DCE.

### 3.3. Population of* Dehalococcoides* and Non-Dehalorespiring Bacteria in the YN3 Metagenome

The metagenome of YN3 indicated the presence of the single known dehalorespiring species,* Dehalococcoides*, in the enrichment culture. The 16S rRNA gene designated as phylotype YN3-01 of the genus* Dehalococcoides *was identical to the 16S rRNA genes of* D. mccartyi *strains CG5, IBARAKI, CBDB1, and DCMB5 in the Pinellas subgroup of* Dehalococcoides *(Figure S2, supplementary material), one of the three subgroups of* Dehalococcoides* proposed by Hendrickson et al. [47]. The 16S rRNA gene of the phylotype YN3-01 was identical to the 16S rRNA genes of strains CG5, CBDB1, IBARAKI, and DCMB5. Strains CBDB1, CG5, and DCMB5 can dechlorinate PCE to TCE or DCE but not DCE and VC [3, 24, 48]. The partial inconsistency between the phylogeny of the 16S rRNA gene and the dechlorination spectrum observed in this study can be explained in two ways: multiple strains of* Dehalococcoides* are present in YN3 or* rdhA*s for* cis*-DCE and VC were horizontally acquired independently of the core genes [49, 50].

**Figure 2:**
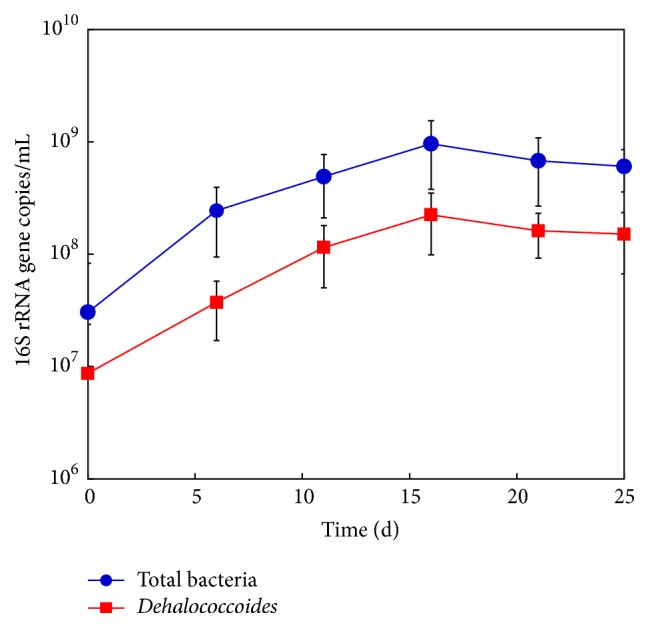
Changes in the population of* Dehalococcoides *and total bacteria in YN3. Error bars represent SDs (*n* = 6).

To confirm PCE-to-ETH dechlorination by* Dehalococcoides* in YN3, the* Dehalococcoides* population was monitored by 16S rRNA gene-based qPCR analysis.  Figure 2 shows the qPCR data for YN3 dechlorinating 70 *μ*M PCE (Figure 1(a)) based on 16S rRNA primers specific for* Dehalococcoides* and total bacteria. YN3 contained 0.86 ± 1.5 × 10^7^ copies of the* Dehalococcoides* 16S rRNA gene/mL of culture at day 0, which increased to 1.6 ± 0.69 × 10^8^ copies/mL at day 21, at which PCE was completely dechlorinated to ETH. Meanwhile, the total bacterial population in YN3 showed 3.0 ± 5.2 × 10^7^ copies/mL at day 0, which increased to 6.7 ± 4.0 × 10^8^ copies/mL at day 21. Throughout the incubation,* Dehalococcoides *species comprised 15–28% of the total bacterial population. Similar growth trends were observed for* Dehalococcoides* and total bacteria in YN3 dechlorinating* cis-*DCE, in which* Dehalococcoides* accounted for 31–45% of the total bacteria (Figure S3, supplementary material).* Bacteroides* species, non-dehalorespiring bacteria that existed together with* Dehalococcoides *species in YN3, reportedly have five 16S rRNA gene copies [51], while* Dehalococcoides* spp. harbor a single copy [49, 52], suggesting that the* Dehalococcoides* population in YN3 was potentially underestimated. YN3 spiked with 200–800 *μ*M PCE showed increasing* Dehalococcoides* copy numbers, as summarized in Table S2 (supplementary material), indicating their dehalorespiring growth at higher concentrations of PCE.

### 3.4. *Dehalococcoides* Metagenome

Sixty-five contigs (1.3 Mbp in total) in the YN3 metagenome were assigned to* Dehalococcoides*. The total size and number of CDSs of the* Dehalococcoides* metagenome from YN3 are similar to those of the other known* Dehalococcoides* isolates (Table S3, supplementary material). Forty-eight out of 65 contigs (104–202,438 bp in length) showed 92–100% similarity to the genomes of* D. mccartyi* strains DCMB5 and CG5, which are isolates belonging to the Pinellas subgroup. The other 17 contigs (129–2,468 bp in length) showed 91–99% similarity to the partial genome sequences of* D. mccartyi* strain VS in the Victoria subgroup. Owing to limited sequencing data, we were not able to deduce whether all of the* Dehalococcoides* contigs were derived from a single strain or from multiple strains.

**Figure 3:**
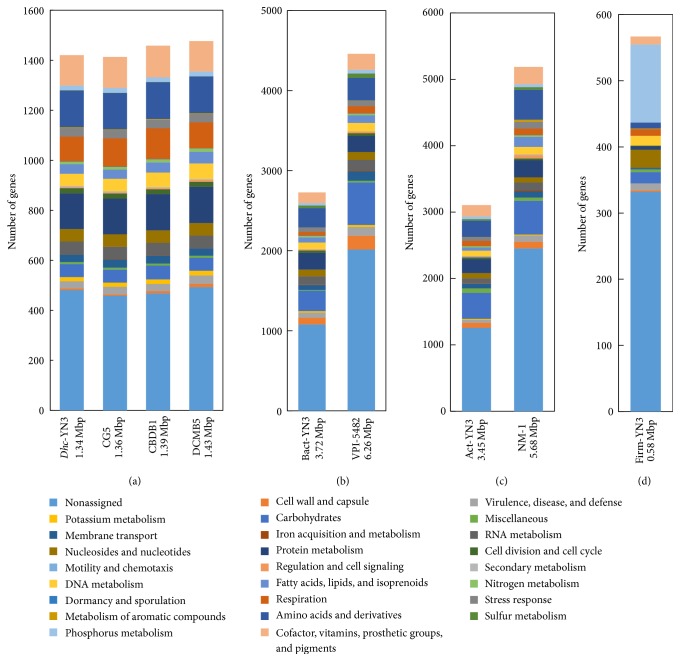
Functional distribution of genes in the YN3 metagenome. (a)* Dehalococcoides* metagenome in YN3 (Dhc-YN3) and* Dehalococcoides* strains CG5, CBDB1, and DCMB5, (b) Bacteroidetes metagenome in YN3 (Bact-YN3) and* Bacteroides thetaiotaomicron *VPI-5482, (c) Actinobacteria metagenome in YN3 (Act-YN3) and* Microlunatus phosphovorus *NM-1, and (d) Firmicutes metagenome in YN3 (Firm-YN3).

The* Dehalococcoides* metagenome was compared to the genomes of strains CG5, CBDB1, and DCMB5, which are the nearest strains according to the above 16S rRNA gene analysis (Figure 3(a)). The genome size (1.34 Mbp) and CDS number (1420) in the* Dehalococcoides* metagenome in YN3 were slightly lower than those of the closest strains, which are 1.39–1.43 Mbp, containing 1413–1477 CDSs. The functional distributions of the genes from the* Dehalococcoides* metagenome and the related strains were very similar, except for minor variation in some categories.

### 3.5. Non-*Dehalococcoides* Metagenome

Genomic fragments of non-*Dehalococcoides* bacteria in YN3 were affiliated to not only Bacteroidetes, but also the phyla Actinobacteria and Firmicutes. In their metagenomes,* rpoB*, encoding the *β*-subunit of RNA polymerase, and the 16S rRNA gene revealed the presence of strains closely related to* Microlunatus phosphovorus* NM-1 of the phylum Actinobacteria (86% of* rpoB* similarity) and* Bacteroides thetaiotaomicron *VPI-5482 of the phylum Bacteroidetes (97% of 16S rRNA gene similarity). Unfortunately, we were unable to compare the Firmicutes metagenome with isolated genomes because of low read numbers and lack of the 16S rRNA or* rpoB* gene in this metagenome (Figure 3(d)).

The Bacteroidetes/Actinobacteria metagenomes had smaller genome sizes and lower total gene numbers than the phylogenetically closest strains (Figures 3(b) and 3(c)). This suggests insufficient sequencing depth to complete the genomes of all members in YN3. In contrast to the overall trend, the Actinobacteria metagenome had 7–19 more genes in the functional categories “motility and chemotaxis” and “miscellaneous,” and the Bacteroidetes metagenome had up to 10 genes more in the categories “dormancy and sporulation,” “secondary metabolism,” “protein metabolism,” “motility and chemotaxis,” and “miscellaneous.” Actinobacteria/Bacteroidetes members with those particular functions are potentially selectively cultivated under dehalorespiration by* Dehalococcoides*.

### 3.6. Reductive Dehalogenase and Associated Genes

**Figure 4:**
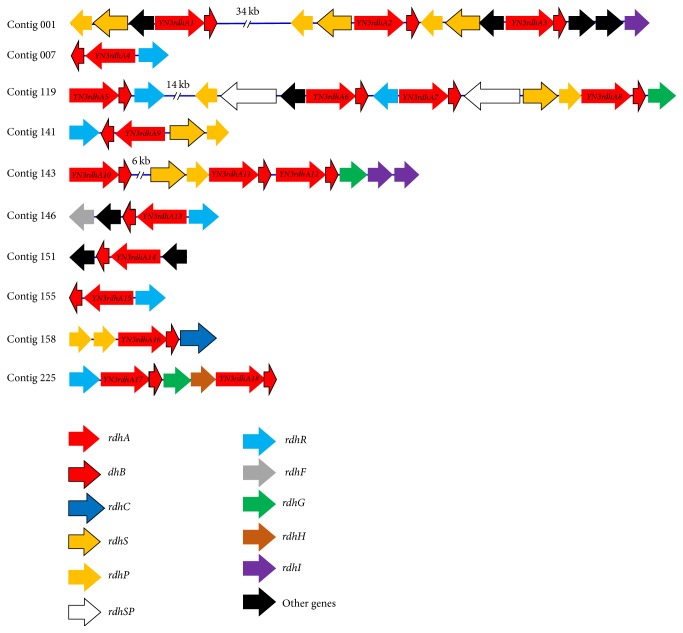
Gene clusters containing reductive dehalogenases in the* Dehalococcoides *metagenome. The predicted encoded proteins for all genes have been described previously [1] and are as follows:* rdhA*: catalytic subunit of Rdh,* rdhB*: membrane anchor protein of Rdh,* rdhC*: protein that has a putative function in regulation or electron transport,* rdhS *and* rdhP*: sensor histidine kinase and response regulator of the two-component regulatory system, respectively,* rdhSP*: hybrid* rdhS *and* rdhP*,* rdhR*: multiple resistance regulator (MarR) regulator,* rdhF*: corrinoid-synthetizing protein,* rdhG*: Rdh-modifying proteolytic protein,* rdhH*: hypothetical protein of unknown function, and* rdhI*: corrinoid-modifying protein.

Eighteen pairs of* rdhA* and* rdhB*, encoding the catalytic unit and membrane anchor protein of Rdh, respectively, were detected in the* Dehalococcoides* metagenome (Figure 4). Most of the predicted RdhA proteins had three conserved motifs of twin-arginine translocation (TAT) leader sequence at the N-terminus and two iron-sulfur-binding motifs near the C-terminus [13], with only YN3RdhA10 lacking the TAT sequence. Most* rdhAB *pairs in the YN3 metagenome were detected with genes of transcription regulators (*rdhS/P* and* rdhR*), suggesting their involvement specific to each* rdhA*. Other associated genes involved in assembling and maturation of RdhA (*rdhF*,* rdhG*,* rdhH*, and* rdhI*) were a minority in the YN3 metagenome, as reported for other isolates of* Dehalococcoides* spp. Surprisingly,* rdhA* encoded in gene clusters lacking those accessory genes is expressed as an active, mature RdhA, as indicated by a previous study [50], which suggests a possible association of these accessory proteins remotely encoded in the genome to multiple RdhAs.

**Figure 5:**
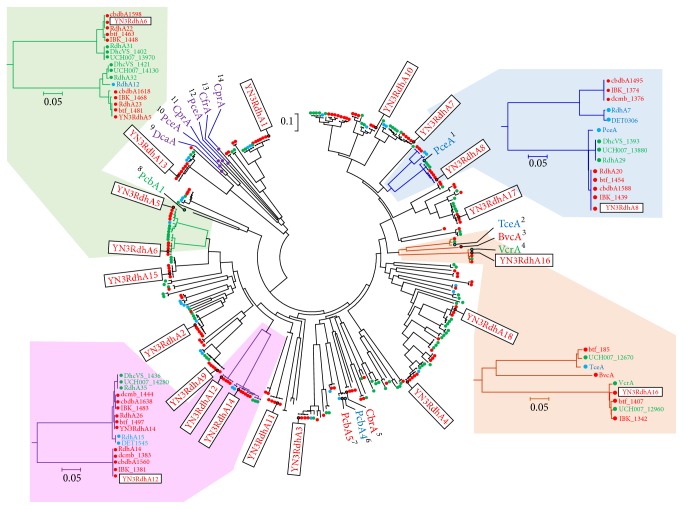
Neighbor-joining tree of RdhAs detected in the YN3–*Dehalococcoides *metagenome. The tree shows the phylogeny of RdhAs detected in YN3–*Dehalococcoides*-metagenome (inside boxes) and other RdhAs from* Dehalococcoides* and non-*Dehalococcoides* dehalorespiring bacteria. Red, blue, and green solid circles indicate RdhAs in the Pinellas, Cornell, and Victoria subgroups of genus* Dehalococcoides*, respectively, while violet indicates those of non-*Dehalococcoides* dehalorespirators. Superscript numbers 1–14 indicate biochemically characterized RdhAs and their substrate(s) as follows: ^1^PceA (AAW40342): RdhA for PCE [17]; ^2^TceA (AAW39060): RdhA for TCE,* cis*-DCE, 1,1-DCE, 1,2-DCA, and 1,2-dibromoethane [18]; ^3^BvcA (ABQ17429): RdhA for* cis*-DCE,* trans*-DCE, 1,1-DCE, 1,2-DCA, and VC [16, 22]; ^4^VcrA (AAQ94119): RdhA for TCE,* cis*-DCE,* trans*-DCE, 1,1-DCE, and VC [15]; ^5^CbrA (CAI82345): RdhA for 1,2,3,4-tetrachlorobenzene, 1,2,3-tetrachlorobenzene and pentachlorobenzene [29]; ^6^PcbA4 (WP_041340852): RdhA for PCB and PCE [24]; ^7^PcbA5 (AII60305): RdhA for PCB and PCE [24]; ^8^PcbA1 (AII58466): RdhA for PCB and PCE [24]; ^9^DcaA (CAJ75430): RdhA for 1,2-DCA [30]; ^10^PceA (CAD28790): RdhA for PCE and TCE [31]; ^11^CprA (AAQ54585): RdhA for 3,5-dichlorophenol, 2,3,5-trichlorophenol, PCP, 2,3,4,5-tetrachlorophenol, 3,4,5-trichlorophenol, 2,4,6-trichlorophenol, 2,4,5-trichlorophenol, and 2,4-dichlorophenol [32]; ^12^PceA (AAC60788): RdhA for PCE and TCE [33]; ^13^CfrA (AFQ20272): RdhA for 1,1,1-trichloroethane and chloroform [34]; ^14^CprA (AAG49543): RdhA for orthochlorophenols [35].

Among the 18 RdhAs in YN3, only YN3RdhA8 and YN3RdhA16 showed significant similarity to the previously experimentally proven CE-dechlorinating RdhAs (Table S4, supplementary material). YN3RdhA8 had 94% amino acid identity with PceA of strain 195, which dechlorinates PCE to TCE [17]. In the phylogenetic tree (Figure 5), YN3RdhA8 formed a cluster with PceA of strain 195 and several RdhAs from PCE-dehalorespiring strains (195, CBDB1, CG1, and BTF08) and non-PCE-dehalorespiring strains VS [53], UCH007 [20], and IBARAKI [21]. YN3RdhA16 showed 97% amino acid similarity with VcrA of strain VS, an RdhA that can dechlorinate* cis*-DCE and VC, and TCE with lower activity [15]. In the phylogenetic tree, YN3RdhA16 clustered with VcrA and RdhAs from* cis*-DCE-to-ETH-dehalorespiring strains UCH007 [20] and IBARAKI [21] and PCE-to-ETH-dechlorinating strain BTF08 [5].

### 3.7. Transcription of* rdhA* in Response to CEs

To identify the* rdhAs* involved in the PCE-to-ETH dechlorination in YN3, RT-qPCR targeting mRNA of all 18* rdhA*s was conducted. The* rdhA*s showing a higher than twofold increase in transcription after spiking of CEs as compared to without spiking by single determination were screened as candidate* rdhA*s for CEs. A first RT-qPCR screening of YN3 grown with* cis*-DCE (Table S5, supplementary material) provided three candidate CEs-responsive* rdhA*s, that is,* YN3rdhA6*,* YN3rdhA12*, and* YN3rdhA16*. Unexpectedly,* YN3rdhA8*, closely related to* pceA* of a known PCE-Rdh, was not transcribed at a significant level, although the starved YN3 spiked with PCE produced TCE.

**Figure 6:**
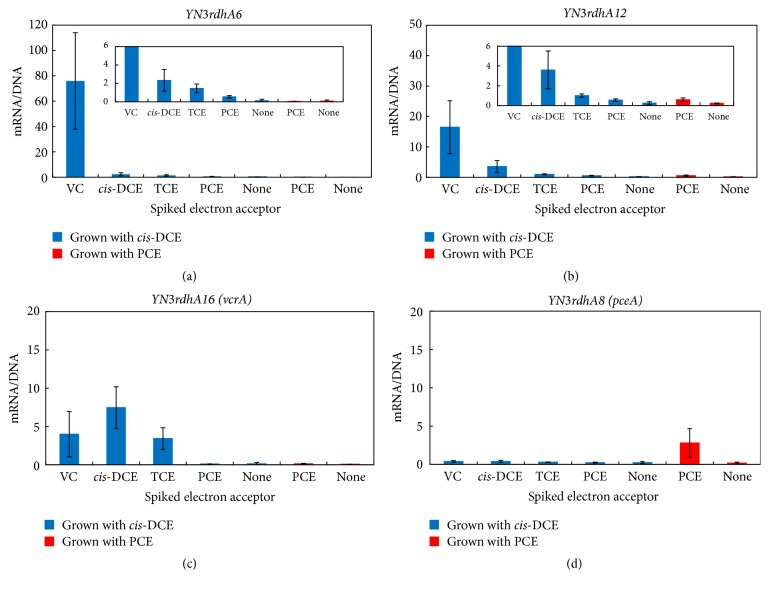
Transcription analysis of* YN3rdhA6* (a),* YN3rdhA12* (b),* YN3rdhA16* (c), and* YN3rdhA8* (d) in YN3 spiked with PCE, TCE,* cis*-DCE, or VC. Error bars represent SDs (*n* = 3). Inserted figures in panels (a) and (b) are enlarged figures. Blue and red represent the transcription of* rdhA* in YN3 grown with* cis*-DCE and PCE, respectively.

Next, the transcription of three* rdhAs* significantly responsive to CE-spiking and* YN3rdhA8* was confirmed in at least triplicate determinations.* YN3rdhA6*,* YN3rdhA12*, and* YN3rdhA16* were confirmed to significantly respond to some of the CEs (Figure 6), while no significant transcription of* YN3rdhA8* was detected. Specifically,* YN3rdhA6* mRNA was greatly increased by spiking of VC (75 ± 38 mRNA copies per gene; Figure 6(a)) and slightly increased by spiking with* cis*-DCE, TCE, and PCE.* YN3rdhA12* responded especially to VC spiking (16 ± 8.6 mRNA copies per gene) and showed a slight response to all other CEs (Figure 6(b)).* YN3rdhA16*, an* rdhA* closely related to* vcrA, *showed increased transcription in response to VC (4.0 ± 2.9 mRNA copies per gene),* cis*-DCE (7.4 ± 2.7 mRNA copies per gene), and TCE (3.4 ± 1.4 mRNA copies per gene) (Figure 6(c)). In this experiment, two* rdhA*s (*YN3rdhA6* and* YN3rdhA12*) that had been never previously identified as* rdhAs* for CEs unexpectedly showed much stronger transcription than the two* rdhAs* corresponding to known CE-RdhAs, that is,* YN3rdhA16 (vcrA)* and* YN3rdhA8 (pceA)*.

The observed marginal increases in* YN3rdhA6* and* YN3rdhA12* mRNA after spiking of PCE and no significant increase in* YN3rdhA8 (pceA)* transcription motivated us to reexamine the transcription by using YN3 grown with PCE. YN3 grown with PCE was starved similar to the above transcription analysis and then spiked with PCE. As a result, an increase in transcription was observed especially for* YN3rdhA8 (pceA)*, with 2.6 ± 1.8 mRNA copies per gene (Table S5 (supplementary material) and Figure 6(d)).

Overall, the results suggested the involvement of four* rdhAs* in different steps of PCE-to-ETH dechlorination.* YN3rdhA8 (pceA)* is seemingly involved in PCE-to-TCE dechlorination, especially under PCE-growing condition. Subsequently,* YN3rdhA16 (vcrA)* is transcribed to respond to the produced TCE and is involved in the dechlorination of TCE to ETH. Besides these known* rdhA*s,* YN3rdhA6* and* YN3rdhA12* were found to be transcribed in the presence of VC and are likely involved in VC dechlorination.

## 4. Discussion

In this study, we successfully obtained a* Dehalococcoides*-dominated PCE-to-ETH-dechlorinating culture, YN3, and revealed that four* rdhA*s are involved in the PCE-to-ETH dechlorination. Particularly, we identified a novel candidate* rdhA, YN3rdhA6*, encoding an RdhA that probably dechlorinates VC, on the basis of its higher transcription than that of other* rdhA*s in response to VC.

To date, only two* rdhAs *encoding RdhAs that dechlorinate VC to ETH, that is,* vcrA* and* bvcA,* have been identified by enzymatic assays [15, 16].* vcrA* was originally identified via amino acid sequencing of the partially purified VcrA [15], while* bvcA* was first suggested as a candidate VC RdhA on the basis of its increased transcription in response to VC [22], and BvcA was recently proven to dechlorinate VC by native gel assay [16]. Both* vcrA* and* bvcA* have been applied as authentic biomarkers to estimate VC dechlorination activity in bioremediation sites [2]. This study suggests the potential presence of multiple VC-reductive dehalogenases in* Dehalococcoides* and an additional VC-reductive dehalogenase-encoding gene,* YN3rdhA6*, far related to* vcrA* and* bvcA*, with 31% translated amino acid similarity. However, some* Dehalococcoides*, either in pure form or in consortia, have* rdhAs* identical or similar (>94% of the translated similarity) to* YN3rdhA6* but are unable to dechlorinate VC (Tables S4 and S6, supplementary material). This contradictory finding might indicate that* YN3rdhA6* responds to VC at the transcriptional level but is not involved in the dechlorination. Additional experiments, including enzymatic assays, are required to further assess whether* YN3rdhA6* encodes a VC-reductive dehalogenase.

The sequences of* YN3rdhA6* and its gene cluster are not unique but are highly conserved in strains of* Dehalococcoides* (Figure S4A, supplementary material). However, the substantial transcriptional response of* YN3rdhA6*-related genes to VC has been missed in previous studies because they generally focused on responses of this gene to PCE [24] (Table S6, supplementary material). The difference in magnitude of the transcriptional response between* YN3rdhA6* and other* rdhA*s can be attributed to a particular transcription regulator, the hybrid protein* (rdhSP)* of sensor histidine kinase (*rdhS)* and response regulator* (rdhP)* of the two-component regulatory system (Figure 4 and Figure S4 (supplementary material)). In the metagenome of* Dehalococcoides* in YN3, another transcription regulator, multiple resistance regulator (MarR) regulator* (rdhR)*, and a gene set of* rdhS* and* rdhP* are frequently observed in multiple Rdh-associated gene clusters and are suggested to function in the transcription regulation of* rdhA*s. The hybrid protein* rdhSP* is particularly detected in only two gene clusters, one of which includes* YN3rdhA6*.

Another* rdhA* that particularly responded to VC spiking,* YN3rdhA12*, is a possible candidate* rdhA* that dechlorinates VC. The gene cluster harboring the gene is conserved in YN3 and* Dehalococcoides* (Figure S4B, supplementary material). The transcription of* rdhA* identical to* YN3rdhA12* has been studied in strain CG5 [24] and a PCE-dechlorinating enrichment culture TUT2264 [54] (Table S6, supplementary material). The* YN3rdhA12*-related* rdhA* of CG5 showed lower transcription than other* rdhA*s in culture, and consequently, this gene has been never focused on as an* rdhA* for CEs in this strain [24]. On the other hand, three* YN3rdhA12*-related* rdhA*s of TUT2264 showed remarkable (>20-fold) increases in transcription in response to spiking of CEs [54]. However, the specific CE affecting transcription was different among* YN3rdhA12 *and the three* rdhA*s in TUT2264. Specifically, like* YN3rdhA12,* one of the* YN3rdhA12*-related* rdhA*s of TUT2264 was increasingly transcribed by addition of VC, while the two other genes were particularly transcribed in the presence of other CEs, but not VC. These results indicated that* YN3rdhA12* and related* rdhA*s can be suggested as* rdhA*s for CEs, although further study is required to identify specifically which CEs can be dechlorinated by the* rdhA*s.

The PCE-to-ETH dechlorination rate in YN3 seemed to be comparatively higher than those previously reported in other PCE-to-ETH-dechlorinating enrichment cultures. The KB-1/PCE culture was reported to dechlorinate 100–300 *μ*M of spiked PCE to ETH within two weeks with the involvement of* Dehalococcoides* and* Geobacter* as dechlorinators [4]; BTF08, a highly enriched culture containing* Dehalococcoides* as a single dehalorespirator, dechlorinated approximately 500 *μ*M PCE to ETH in approximately 100 days [5]; AMEC-4P culture dechlorinated 2 mM PCE to ETH within 143 days, also with* Dehalococcoides* and* Geobacter* as dechlorinators [6]. Although the dechlorination rates of those microbial communities are variable depending on the culture conditions, YN3 can be suggested as a promising consortium for application in the bioaugmentation of environments contaminated with CEs.

In contrast to the relatively higher dechlorinating activity in YN3, metagenome analysis of YN3 indicated no apparent uniqueness in the genome of* Dehalococcoides* in this culture. Specifically,* Dehalococcoides* in YN3 showed 16S rRNA gene sequences identical to those of strains CG5, CBDB1, IBARAKI, and DCMB5 (Figure S2, supplementary material) in the Pinellas subgroup of* Dehalococcoides*. Moreover, all the predicted RdhAs detected in YN3 showed 98–100% similarity with some of the RdhAs in those strains (Table S4, supplementary material). Therefore, we suggest that the high dechlorination activity in YN3 is attributable to other mechanisms.

A plausible explanation for the high dechlorination activity of YN3 is the constitution of its microbial community. The metagenomic analysis indicated the coexistence of members of the phyla Bacteroidetes, Firmicutes, and Actinobacteria. Particularly, a 16S rRNA gene-based phylotype of* Bacteroides* might be a new species of* Bacteroides*, on the basis of the 16S rRNA gene similarity (97%) with known strains. Additionally, members of* Bacteroides* have been frequently detected in dehalorespiring cultures together with* Dehalococcoides *[11, 55, 56]. To date, coexisting bacteria belonging to diverse bacterial phyla have been reported to support* Dehalococcoides* by supplementation of growth factors [8, 9], removal of toxic substances [12], and scavenging the harmful oxygen [57]. Considering the variety in function and phylogeny of non-dechlorinating bacteria that support dechlorination, the non-dechlorinating bacteria in YN3 also possibly contribute to enhancing the dechlorination activity of YN3. Additional experiments will be necessary to prove their contribution to dechlorination by YN3.

## 5. Conclusion

A* Dehalococcoides*-dominated enrichment culture, YN3, was newly established from a CE-contaminated river sediment and efficiently dechlorinated 800 *μ*M PCE to ETH within 14 days. The high dechlorination rate was attributed to the presence of non-dehalorespiring bacteria of phyla Actinobacteria and Firmicutes and a new species of the phylum Bacteroidetes. Transcription analysis suggested the involvement of four* rdhAs* in the PCE-to-ETH dechlorination in YN3. Among these four* rdhA*s, two were closely related to well-known* rdhA*s involved in the dechlorination of CEs,* pceA*, and* vcrA*. The other two* rdhAs*,* YN3rdhA6* and* YN3rdhA12*, can be novel candidate* rdhA*s encoding VC-reductive dehalogenases, on the basis of their significant increase in transcription in response to VC.

## Supplementary Material

This supplementary material provides an additional information regarding this study such as the primers used, 16S rRNA gene phylogenetic tree for the detected *Dehalococcoides*, *cis*-DCE dechlorination, characteristics of the assembled *Dehalococcoides*-metagenome, similarity comparison of the detected RdhAs with previously identified RdhAs and gene cluster of some detected rdhA genes.
