# Supplementary material for: Identification of Multiple Dehalogenase Genes Involved in Tetrachloroethene-to-Ethene Dechlorination in a Dehalococcoides-Dominated Enrichment Culture
Source: Biomed Res Int. 2017 Aug 15;2017:9191086. doi: 10.1155/2017/9191086 (PMC5574268; doi:10.1155/2017/9191086)
Supplement: Supplementary file 1 — This supplementary material provides an additional information regarding this study such as the primers used, 16S rRNA gene phylogenetic tree for the detected Dehalococcoides, cis-DCE dechlorination, characteristics of the assembled Dehalococcoides-metagenome, similarity comparison of the detected RdhAs with previously identified RdhAs and gene cluster of some detected rdhA genes. [file 9191086.f1.doc]

**Supplementary Information**

**Identification of Multiple Dehalogenase Genes Involved in Tetrachloroethene-to-Ethene Dechlorination in a *Dehalococcoides*-Dominated Enrichment Culture**

Mohamed Ismaeil^1^, Naoko Yoshida^2*^**,** Arata Katayama^1,3,4^

^1^ Department of Environmental Engineering and Architecture, Graduate School of Environmental Studies, Nagoya University, Chikusa, Nagoya 464-8603, Japan

^2^ Department of Civil Engineering, Nagoya Institute of Technology, Showa, Nagoya 466-8555, Japan

^3^ Department of Civil Engineering, Graduate School of Engineering, Nagoya University, Chikusa, Nagoya 464-8603, Japan

^4^ Institute of Materials and Systems for Sustainability (IMaSS), Nagoya University, Chikusa, Nagoya 464-8603, Japan

*Corresponding author: yoshida.naoko@nitech.ac.jp

**Supplementary Table S1:** list of primers used in this study

| **Primer** | **Target** | **Direction** | **Sequence (5´ to 3´)** | **Reference** |
| --- | --- | --- | --- | --- |
| 341F | Bacterial 16S rRNA gene | Forward | CCTACGGGAGGCAGCAG | 1 |
| 518R |  | Reverse | ATTACCGCGGCTGGCTGG |  |
| DhcF50 | 16S rRNA gene of  *Dehalococcoides* | Forward | CCTTATGCATGCAAGTCGAA | This study |
| DhcR155 |  | Reverse | ACCACATGCGGTATTACCTTC |  |
| F640 | *YN3rdhA1* | Forward | TATACTACCGGTCACGCCAA | This study |
| R741 |  | Reverse | CGGTAAGTGCATTCAGGGTA |  |
| F117 | *YN3rdhA2* | Forward | AGCTGTCTCTGCGCCTGTAG | This study |
| R219 |  | Reverse | TCCACGAATTGTCCGAACGC |  |
| F3 | *YN3rdhA3* | Forward | GCAAGGATTCCATTCTGCTT | This study |
| R107 |  | Reverse | GGAAGCACCTTCATCCAAAT |  |
| F1143 | *YN3rdhA4* | Forward | CCAGAGCTGGGCTGATGACA | This study |
| R1239 |  | Reverse | CGCTGGCACAAGGCGTAAAT |  |
| F926 | *YN3rdhA5* | Forward | TGGTTACTGACCTGCCGCTT | This study |
| R1010 |  | Reverse | ACACTGGGCGGGACATTCAT |  |
| F1050 | *YN3rdhA6* | Forward | TGAGCCTACTCCGCCCATTG | This study |
| R1136 |  | Reverse | GGGCTCATGGTCAGTGGGAA |  |
| F743 | *YN3rdhA7* | Forward | ACGTAGACGAAGCGGCTGAA | This study |
| R828 |  | Reverse | GGCGTCTGGTGGACTCGTAA |  |
| F600 | *YN3rdhA8* | Forward | GGGTACTCCCGAAGATAACCT | This study |
| R706 |  | Reverse | CCGTACTGGTCAACGGTAAA |  |
| F1323 | *YN3rdhA9* | Forward | CCTTTCCACCACCGGCATTT | This study |
| R1417 |  | Reverse | AAGCCGTATCTGGGCAGGTC |  |
| F616 | *YN3rdhA10* | Forward | CAGGATGTTGGTTGTGCCGAA | This study |
| R705 |  | Reverse | CAATCTGTTTGCCGCCTGCT |  |
| F373 | *YN3rdhA11* | Forward | TATGGCTGGCAGGCAGGTTT | This study |
| R460 |  | Reverse | CTGCCATTCCAAGCAGGCAA |  |
| F1248 | *YN3rdhA12* | Forward | AGGCTTCTGGCCTGACATGG | This study |
| R1345 |  | Reverse | TGCATCATGGCGGCTTTGTC |  |
| F116 | *YN3rdhA13* | Forward | ACTCTGTGGGCGGCATACAT | This study |
| R200 |  | Reverse | ATAGCCGACTGCCGGGTTAC |  |
| F381 | *YN3rdhA14* | Forward | GGGTACTTCTCCCGGTTGGG | This study |
| R480 |  | Reverse | CCGCCCTGAGCATACGTGAA |  |
| F128 | *YN3rdhA15* | Forward | CGCCTAAGGCTGAATGGAAACG | This study |
| R220 |  | Reverse | CTGTCTGACGGGCATCCCA |  |
| F459 | *YN3rdhA16* | Forward | GCACGGACTATTCCAACCTT | This study |
| R565 |  | Reverse | TCTGGAGTACCCTCCCATTT |  |
| F55 | *YN3rdhA17* | Forward | TTAGCAGGCGCGGGAGTAG | This study |
| R148 |  | Reverse | TGCTTAACCCACCAGGGCAT |  |
| F2 | *YN3rdhA18* | Forward | TGAAGGGACTGGGTCTGGCT | This study |
| R93 |  | Reverse | GGGCGACTAAGTGTTGCCTGA |  |

**Supplementary Table S2.** Populations of *Dehalococcoides* and total bacteria in the YN3 that completely dechlorinated PCE at higher concentration.

| **PCE conc. (μM)** | **Time after additional spiking of PCE (d)** | ***Dehalococcoides***  **(16S rRNA gene copies/mL)** | **Total bacteria**  **(16S rRNA gene copies/mL)** |
| --- | --- | --- | --- |
| None |  | 5.7 ± 1.9 ×10^7^ | 2.9 ± 1.1 ×10^8^ |
| 200 | 10 | 4.6 ± 0.7 ×10^8^ | 1.0 ± 0.27 ×10^9^ |
| 400 | 12 | 5.1 ± 0.4 ×10^8^ | 8.6 ± 2.1 ×10^8^ |
| 600 | 14 | 6.4 ± 1.3 ×10^8^ | 1.0 ± 0.26 ×10^9^ |
| 800 | 14 | 9.5 ± 2.4 ×10^8^ | 1.4 ± 0.52 ×10^9^ |

**Supplementary Table S3.** Overall characteristics of the YN3-*Dehalococcoides*-metagenome and comparison with genomes of other strains of *Dehalococcoides*

| **Strain or consortium** | **Size** | **% G+C** | **CDS** | **rRNAs** | **tRNAs** | **Predicted *rdhAs*** | **Reference** |
| --- | --- | --- | --- | --- | --- | --- | --- |
| YN3 | 1,346,295 | 47.2 | 1,420 | 3 | 46 | 18 | This study |
| CBDB1 | 1,395,502 | 47.0 | 1,458 | 3 | 47 | 32 | 2 |
| DCMB5 | 1,431,902 | 47.1 | 1,477 | 3 | 46 | 23 | 3 |
| CG5 | 1,362,151 | 47.2 | 1,413 | 3 | 47 | 26 | 4 |
| BTF08 | 1,452,335 | 47.3 | 1,529 | 3 | 46 | 20 | 3 |
| BAV1 | 1,341,892 | 47.2 | 1,371 | 3 | 48 | 11 | 5 |
| CG1 | 1,486,678 | 46,9 | 1,557 | 3 | 49 | 35 | 4 |
| VS | 1,413,462 | 47.3 | 1,442 | 3 | 50 | 36 | 5 |
| IBARAKI | 1,451,062 | 47.0 | 1,516 | 3 | 47 | 28 | 6 |
| UCH007 | 1,473,548 | 46.9 | 1,509 | 3 | 47 | 29 | 7 |
| CG4 | 1,382,308 | 48.7 | 1,421 | 3 | 47 | 15 | 4 |
| 195 | 1,469,720 | 48.9 | 1,591 | 3 | 46 | 17 | 8 |

***Supplementary Table S4.*** *Comparison of dechlorination activity for CEs in YN3 culture and RdhAs in the YN3-Dehalococcoidess-metagenome with those of other strains or consortia of* Dehalococcoides.

| Strain or consortium | YN3 | IBARAKI | UCH007 | BTF08 | DCMB5 | BAV1 | VS | CBDB1 | 195 | CG1 | CG4 | CG5 |
| --- | --- | --- | --- | --- | --- | --- | --- | --- | --- | --- | --- | --- |
| Dechlorinated CEs | PCE  TCE *cis*-DCE  VC | *cis*-DCE  VC | TCE  *cis*-DCE  VC | PCE  TCE  DCE  VC | PCE | (PCE)^a^  (TCE)^a^  *cis*-DCE  VC | TCE  *cis*-DCE  VC | PCE  TCE | PCE  TCE  *cis*-DCE  (VC) ^a^ | PCE  TCE | PCE  TCE | PCE  TCE |
| CEs are not dechlorinated |  |  |  |  | TCE  DCE  VC |  | PCE | *cis*-DCE  VC |  | *cis*-DCE  VC | *cis*-DCE  VC | *cis*-DCE  VC |
| RdhAs showed ≥85 % similarity to those in *Dehalococcoides*-metagenome of YN3 (similarity, %) | YN3RdhA1 | IBK_0226 (99) | UCH007_02120 (95) | btf_121  (99) | dcmb_184 (99) | DehaBAV1_0173 (99) | DhcVS_169 (96) | cbdbA187  (100) | DET0180 (95) | RD5 (96) | RD4 (95) | RD3 (100) |
|  | YN3RdhA2 | IBK_0275 (99) | UCH007_13410 (96) |  | dcmb_235 (99) | DehaBAV1_0121 (99) | DhcVS_1353 (96) | cbdbA238  (99) | DET0302 (95) |  |  | RD4 (100) |
|  | YN3RdhA3 | IBK_0283 (99) |  |  | dcmb_240 (99) |  |  | cbdbA243  (89) | DET0235 (94) | RD1 (93) | RD5 (86) | RD5 (99) |
|  | YN3RdhA4 | IBK_1434 (99) | UCH007_13710 (88) | btf_1440 (99) |  |  | DhcVS_1375  (87) | cbdbA1575  (100) | DET1519 (92) | RD5 (87) |  | RD17 (100) |
|  | YN3RdhA5 | IBK_1468 (99) | UCH007_14130 (95) | btf_1481 (99) |  |  | DhcVS_1421  (95) | cbdbA1618  (99) |  | RD32 (95) |  | RD23 (100) |
|  | YN3RdhA6^c^ | IBK_1448 (99) |  | btf_1463 (99) |  |  | DhcVS_1402  (94) | cbdbA1598  (100) |  | RD31 (95) |  | RD22 (100) |
|  | YN3RdhA7 | IBK_1445 (99) | UCH007_13940 (97) | btf_1460 (97) |  |  | DhcVS_1316  (94) | cbdbA1595  (99) | DET1535 (93) | RD22 (94) | RD11 (94) | RD21 (99) |
|  |  |  |  |  |  |  | DhcVS_1399  (96) |  |  | RD30 (96) |  |  |
|  | YN3RdhA8^c^ | IBK_1439 (100) | UCH007_13880 (96) | btf_1454 (100) |  |  | DhcVS_1393  (96) | cbdbA1588  (100) | DET0318 (PceA)^b^ (94) | RD29 (96) |  | RD20 (100) |
|  | YN3RdhA9 | IBK_1477 (100) | UCH007_14220 (98) | btf_1491 (100) | dcmb_1438(99) |  | DhcVS_1430  (98) | cbdbA1627  (99) | DET1538 (87) | RD34 (98) | RD14 (87) | RD25 (100) |
|  | YN3RdhA10 |  | UCH007_13740 (93) |  |  |  |  | cbdbA1570  (99) | DET1522 (94) | RD26 (93) |  | RD16 (99) |
|  | YN3RdhA11 | IBK_1383 (100) |  |  | dcmb_1385(100) |  |  | cbdbA1563  (100) |  |  |  | RD11 (100) |
|  | YN3RdhA12^c^ | IBK_1381 (100) |  |  | dcmb_1383(100) |  |  | cbdbA1560  (100) |  |  |  | RD14 (100) |
|  | YN3RdhA13 | IBK_0998 (99) | UCH007_13480 (96) | btf_1057 (99) | dcmb_1041(99) | DehaBAV1_0988 (99) |  | cbdbA1092  (100) | DET1171 (95) |  | RD10 (95) | RD6 (99) |
|  | YN3RdhA14 | IBK_1483 (99) | UCH007_14280 (99) | btf_1497 (99) | dcmb_1444(99) |  | DhcVS_1436  (98) | cbdbA1638  (99) | DET1545 (94) | RD35 (98) | RD15 (94) | RD26 (100) |
|  | YN3RdhA15 | IBK_1313 (100) | UCH007_12960 (97) |  | dcmb_134 (100) |  | DhcVS_1263  (90) | cbdbA1455  (99) |  | RD8 (90) |  | RdhA8 (100) |
|  | YN3RdhA16^c^ | IBK_1342 (98) | UCH007_13830 (95) | btf_1407 (97) |  |  | DhcVS_1291  ( VcrA)^b^ (97) |  |  |  |  |  |
|  | YN3RdhA17 |  | UCH007_13640 (92) | btf_1449 (99) |  |  | DhcVS_1387  (95) | cbdbA1582  (100) |  | RD28 (95) |  | RD19 (100) |
|  | YN3RdhA18 |  | UCH007_13790 (94) | btf_1443 (99) |  |  | DhcVS_1383  (94) | cbdbA1578  (99) |  | RD27 (95) |  | RD18 (100) |
| RdhAs showed <85 % similarity to those in *Dehalococcoides*-metagenome of YN3 |  | IBK_0161  IBK_1311  IBK_1358  IBK_1368  IBK_1374  IBK_1409  IBK_1411  IBK_1415  IBK_1418  IBK_1420  IBK_1423  IBK_1427  IBK_1434  IBK_1474 | UCH007_00760  UCH007_00810  UCH007_08570  UCH007_09870  UCH007_09900  UCH007_09930  UCH007_12300  UCH007_12320  UCH007_12670  UCH007_13520  UCH007_13580  UCH007_13610  UCH007_13670  UCH007_13970  UCH007_14190 | btf_1393  btf_1420  btf_1412  btf_1436  btf_1446  btf_1488  btf_1497 | dcmb_81  dcmb_86  dcmb_91  dcmb_113  dcmb_120  dcmb_1041  dcmb_1339  dcmb_1362  dcmb_1366  dcmb_1370  dcmb_1376  dcmb_1428  dcmb_1430  dcmb_1434 | DehaBAV1_0104  DehaBAV1_0112  DehaBAV1_0119  DehaBAV1_0276  DehaBAV1_0281  DehaBAV1_0284  DehaBAV1_0296  DehaBAV1_0847-(BvcA) ^b^ | DhcVS_1427  DhcVS_1260-  DhcVS_1324  DhcVS_1316-  DhcVS_1336  DhcVS_96  DhcVS_1340  DhcVS_1329  DhcVS_1320  DhcVS_99  DhcVS_1342  DhcVS_1344  DhcVS_88  DhcVS_82  DhcVS_104  DhcVS_1327  DhcVS_1314  DhcVS_1360  DhcVS_1349  DhcVS_1378 | cbdbA1453  cbdbA1624  cbdbA1550  cbdbA1495  cbdbA1508  cbdbA96  cbdbA1535  cbdbA1546  cbdbA1542  cbdbA1491  cbdbA88  cbdbA80  cbdbA1503  cbdbA1539  cbdbA84 (CbrA) ^b^ | DET0079  (TceA) ^b^  DET0173  DET0306  DET0311  DET0876-  DET1528  DET1559 | RD2 ,  RD3  RD4  RD6  RD7  RD9  RD10  RD11  RD12  RD13  RD14  RD15  RD16  RD17 (PcbA1) ^b^  RD18  RD19  RD20  RD21  RD23  RD24  RD33 | RD1 (PcbA4)^b^  RD2  RD3  RD6  RD7  RD8  RD9  RD12  RD13 | RD1  (PcbA5)^b^  RD2  RD7  RD9  RD10  RD12  RD13  RD15  RD24 |

^a^:CEs dechlorinated through cometabolic reaction are shown in parentheses, ^b^: The functionally identified RdhAs are shown in blue, ^d^: RdhA expected to be involved in the PCE-to-ETH dechlorination in YN3are shown in red.

**Supplementary Table S5:** The change in transcription of YN3*rdhA1-18* in response to spiking of CEs

|  | **YN3 grown with *cis*-DCE** | | | | **YN3 grown with PCE** |
| --- | --- | --- | --- | --- | --- |
| **Spiked CEs** | **VC** | ***cis*-DCE** | **TCE** | **PCE** | **PCE** |
| *YN3rdhA1* | - | - | - | - | - |
| *YN3rdhA2* | - | - | - | - | - |
| *YN3rdhA3* | - | - | - | - | - |
| *YN3rdhA4* | - | - | - | - | - |
| *YN3rdhA5* | - | - | - | - | - |
| *YN3rdhA6* | + | + | + | - | - |
| *YN3rdhA7* | - | - | - | - | - |
| *YN3rdhA8* | - | - | - | - | + |
| *YN3rdhA9* | - | - | - | - | - |
| *YN3rdhA10* | - | - | - | - | - |
| *YN3rdhA11* | - | - | - | - | - |
| *YN3rdhA12* | + | + | + | - | - |
| *YN3rdhA13* | - | - | - | - | - |
| *YN3rdhA14* | - | - | - | - | - |
| *YN3rdhA15* | - | - | - | - | - |
| *YN3rdhA16* | + | + | + | - | - |
| *YN3rdhA17* | - | - | - | - | - |
| *YN3rdhA18* | - | - | - | - | - |

+ indicates more than 2 fold increase in transcription of *rdhA* to that without spiking

- indicates transcription of *rdhA* less than 2 fold to that without spiking

**Supplementary Table S6:** Comparison of transcribed *rdhAs* genes in response to CEs in *Dehalococcoides*s-metagenome with those of other consortium or strains of *Dehalococcoides.*

| Strain or consortium | YN3 | TUT2264 | KB1 | BAV1 | DCMB5 | VS | CBDB1 | IBARAKI | BTF08 | 195 | CG1 | CG5 |
| --- | --- | --- | --- | --- | --- | --- | --- | --- | --- | --- | --- | --- |
| RdhAs showed ≥85 % similarity to those in *Dehalococcoides*-metagenome of YN3 (similarity, %) ^b^ | YN3RdhA1 |  |  | DehaBAV1_0173 (99) | dcmb_184 (99) | DhcVS_169 (96) | cbdbA187  (100) | IBK_0226 (99) | btf_121  (99) | DET0180 (95) | RD5 (96) | RD3 (100) |
|  | YN3RdhA222 |  |  | DehaBAV1_0121 (99) | dcmb_235 (99) | DhcVS_1353 (96) | cbdbA238  (99) | IBK_0275 (99) |  | DET0302 (95) |  | RD4 (100) |
|  | YN3RdhA3 |  |  |  | dcmb_240 (99) |  | cbdbA243  (89) | IBK_0283 (99) |  | DET0235 (94) | RD1 (93) | RD5 (99) |
|  | YYN3RdhA4 |  |  |  |  | DhcVS_1375  (87) | cbdbA1575  (100) | IBK_1434 (99) | btf_1440 (99) | DET1519 (92) | RD25 (87) | RD17 (100) |
|  | YYN3RdhA5 | RdhA8  (99) |  |  |  | DhcVS_1421  (95) | cbdbA1618  (99) | IBK_1468 (99) | btf_1481 (99) |  | RdD32 (95) | RD23 (100) |
|  | YYN3RdhA6 |  | RdhA2(99) |  |  | DhcVS_1402  (94) | cbdbA1598  (100) | IBK_1448 (99) | btf_1463 (99) |  | RD31 (95) | RD22 (100) |
|  | YYN3RdhA7 |  | RdhA10(99) |  |  | DhcVS_1316  (94) | cbdbA1595  (99) | IBK_1445 (99) | btf_1460 (97) | DET1535 (93) | RD22 (94) | RD21 (99) |
|  |  |  |  |  |  | DhcVS_1399  (96) |  |  |  |  | RD30 (96) |  |
|  | YYN3RdhA8 |  | RdhA13(100) |  |  | DhcVS_1393  (96) | cbdbA1588  (100) | IBK_1439 (100) | btf_1454 (100) | DET0318 (PceA)^c^ (94) | RD29 (96) | RD20 (100) |
|  | YYN3RdhA9 |  | RdhA4(99) |  | dcmb_1438(99) | DhcVS_1430  (98) | cbdbA1627  (99) | IBK_1477 (100) | btf_1491 (100) | DET1538 (87) | RD34 (98) | RD25 (100) |
|  | YYN3RdhA100 |  |  |  |  |  | cbdbA1570  (99) |  |  | DET1522 (94) | RD26 (93) | RD16 (99) |
|  | YYN3RdhA11 |  |  |  | dcmb_1385(100) |  | cbdbA1563  (100) | IBK_1383 (100) |  |  |  | RD11 (100) |
|  | YYN3RdhA12 | RdhA3(98)  RdhA4(100)  RdhA5(100) |  |  | dcmb_1383(100) |  | cbdbA1560  (100) | IBK_1381 (100) |  |  |  | RD14 (100) |
|  | YYN3RdhA13 |  |  | DehaBAV1_0988 (99) | dcmb_1041(99) |  | cbdbA1092  (100) | IBK_0998 (99) | btf_1057 (99) | DET1171 (95) |  | RD6 (99) |
|  | YYN3RdhA14 | RdhA2(99) | RdhA5(99) |  | dcmb_1444(99) | DhcVS_1436  (98) | cbdbA1638  (99) | IBK_1483 (99) | btf_1497 (99) | DET1545 (94) | RD35 (98) | RD26 (100) |
|  | YYN3RdhA15 | RdhA6(97)  RdhA7(99) | RdhA13(99) |  | dcmb_134 (100) | DhcVS_1263  (90) | cbdbA1455  (99) | IBK_1313 (100) |  |  | RD8 (90) | RD8 (100) |
|  | YN3RdhA16 |  | RdhA14(98) |  |  | DhcVS_1291  ( VcrA)^c^ (97) |  | IBK_1342 (98) | btf_1407 (97) |  |  |  |
|  | YYN3RdhA17 |  |  |  |  | DhcVS_1387  (95) | cbdbA1582  (100) |  | btf_1449 (99) |  | RD28 (95) | RD19 (100) |
|  | YN3RdhA18 | RdhA1(100) | RdhA7(99) |  |  | DhcVS_1383  (94) | cbdbA1578  (99) |  | btf_1443 (99) |  | RD27 (95) | RD18 (100) |
| RdhAs showed <85 % similarity to those in *Dehalococcoides*-metagenome of YN3 |  |  | RdhA1  RdhA3  RdhA6  RdhA8  RdhA9  RdhA11  RdhA12 | DehaBAV1_0104  DehaBAV1_0112  DehaBAV1_0119  DehaBAV1_0276  DehaBAV1_0281  DehaBAV1_0284  DehaBAV1_0296  DehaBAV1_0847-(BvcA) ^c^ | dcmb_81  dcmb_86  dcmb_91  dcmb_113  dcmb_120  dcmb_1041  dcmb_1339  dcmb_1362  dcmb_1366  dcmb_1370  dcmb_1376  dcmb_1428  dcmb_1430  dcmb_1434 | DhcVS_1427  DhcVS_1260  DhcVS_1324  DhcVS_1316  DhcVS_1336  DhcVS_96  DhcVS_1340  DhcVS_1329  DhcVS_1320  DhcVS_99  DhcVS_1342  DhcVS_1344  DhcVS_88  DhcVS_82  DhcVS_104  DhcVS_1327  DhcVS_1314  DhcVS_1360  DhcVS_1349  DhcVS_1378 | cbdbA1453  cbdbA1624  cbdbA1550  cbdbA1495  cbdbA1508  cbdbA96  cbdbA1535  cbdbA1546  cbdbA1542  cbdbA1491  cbdbA88  cbdbA80  cbdbA1503  cbdbA1539  cbdbA84 (CbrA) ^c^ | IBK_0161  IBK_1311  IBK_1358  IBK_1368  IBK_1374  IBK_1409  IBK_1411  IBK_1415  IBK_1418  IBK_1420  IBK_1423  IBK_1427  IBK_1434  IBK_1474 | btf_1393  btf_1420  btf_1412  btf_1436  btf_1446  btf_1488  btf_1497 | DET0079  (TceA) ^c^  DET0173  DET0306  DET0311  DET0876-  DET1528  DET1559 | RD2 ,  RD3  RD4  RD6  RD7  RD9  RD10  RD11  RD12  RD13  RD14  RD15  RD16  RD17 (PcbA1) ^c^  RD18  RD19  RD20  RD21  RD23  RD24  RD33 | RD1  (PcbA5)^c^  RD2  RD7  RD9  RD10  RD12  RD13  RD15  RD24 |

Transcibed with (PCE or TCE) Transcibed with (*cis*-DCE) Transcibed with (PCE)

Transcibed with (VC) Not transcibed with any CEs

**Supplementary Figure S1.** Reductive dechlorination of *cis*-DCE to ETH in YN3. Error bars represent SDs (n = 3)

**Supplementary Figure S2.** Neighbor-joining tree of *Dehalococcoide*s species based on the 16S rRNA gene. P, V, and C represent taxonomic subgroups of *Dehalococcoides*, where (P) = Pinellas, (C) = Cornell, and (V) = Victoria. The Genebank accession numbers are shown in parentheses. The bar represents 0.05 substitutions per 100 bp.

**Supplementary Figure S3:** Changes in populations of *Dehalococcoides* and total bacteria in YN3 dechlorinating *cis*-DCE to ETH. Error bars represent SDs (n = 3).


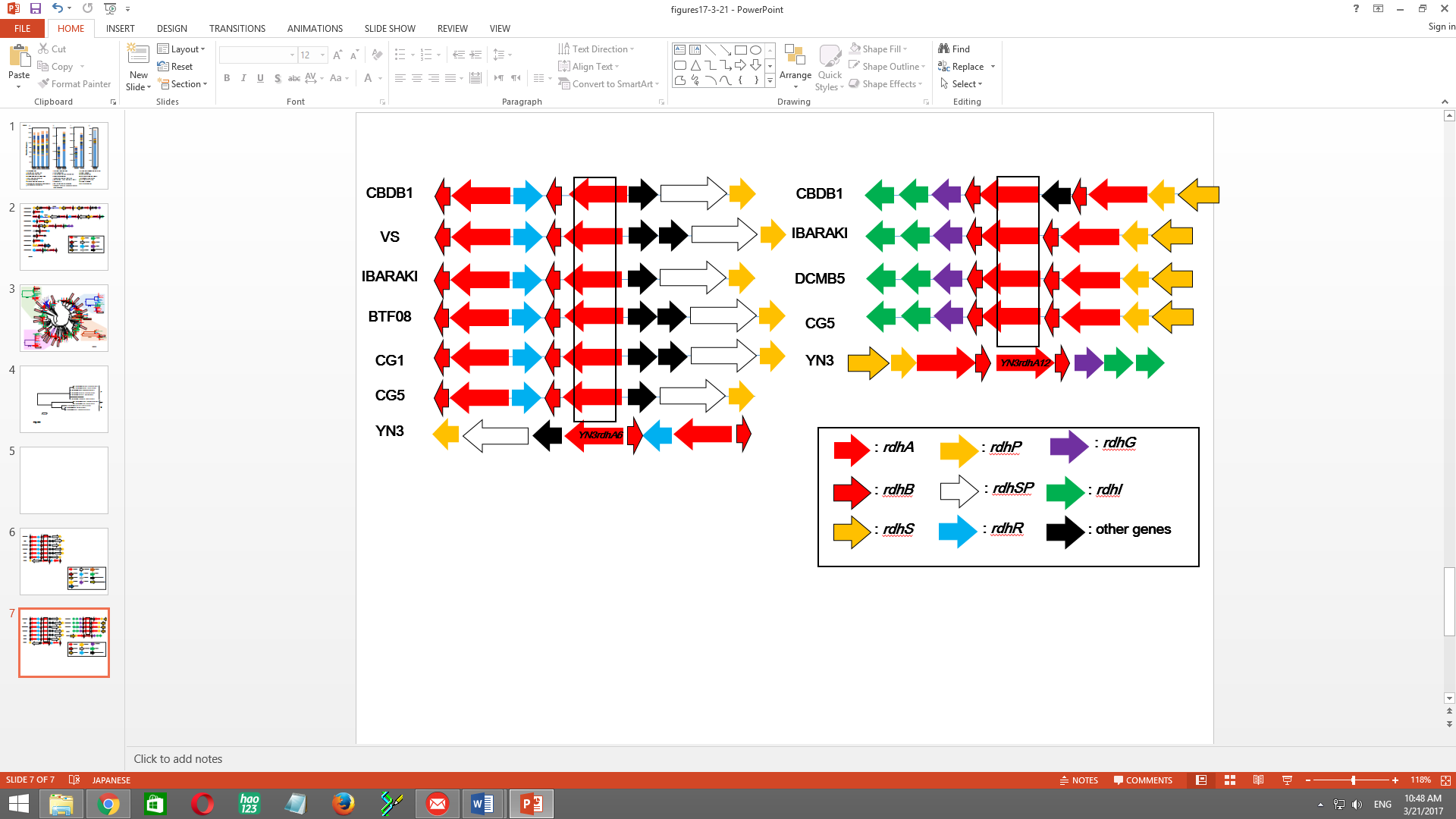


A

B

**Supplementary Figure S4**: Comparsion of gene cluster for *YN3rdhA6* (A) and *YN3rdhA12* (B) and their related *rdhAs* gene cluster from *Dehalococcoides.* Rectangles show the *rdhAs* related to *YN3rdhA6* or *YN3rdhA12.* The predicted encoded proteins for all genes have been described previously [1], and are as follows; *rdhA*: catalytic subunit of Rdh, *rdhB*: membrane anchor protein of Rdh, *rdhS*and*rdhP*: sensor histidine kinase and response regulator of the two component regulatory system, respectively, *rdhSP*: hybrid *rdhS*and*rdhP*, *rdhR*: multiple resistance regulator (MarR) regulator, *rdhG*: Rdh-modifying proteolytic protein and*rdhI*: corrinoid-modifying protein.

**References**

[1] G. Muyzer, E.C. de Waal, and A.G. Uitterlinden,“Profiling of complex microbial populations by denaturing gradient gel electrophoresis analysis of polymerase chain reaction-amplified genes coding for 16S rRNA.” *Applied and Environmental Microbiology,* vol. 59, no. 10, pp. 695–700, 1993.

[2] M. Kube, A. Beck, S.H. Zinder, H. Kuhl, R. Reinhardt, and L. Adrian, “Genome sequence of the chlorinated compound-respiring bacterium *Dehalococcoides* species strain CBDB1.” *Nature Biotechnology,* vol. 23, no. 10, pp. 1269–1273, 2005.

[3] M. Pöritz, T. Goris, T. Wubet, M.T. Tarkka, F. Buscot, I. Nijenhuis, U. Lechner, and L. Adrian, “Genome sequences of two dehalogenation specialists *- Dehalococcoides mccartyi* strains BTF08 and DCMB5 enriched from the highly polluted Bitterfeld region.” *FEMS Microbiology Letters,*  vol. 343, no. 2, pp. 101–104, 2013.

[4] S. Wang, K.R. Chng, A. Wilm, S. Zhao, K.-L. Yang, N. Nagarajan, and J. He “Genomic characterization of three unique *Dehalococcoides* that respire on persistent polychlorinated biphenyls.” *Proceedings of the National Academy of Sciences U. S. A,* vol. 111, no. 33, pp. 12103–12108, 2014.

[5] P.J. McMurdie, S.F. Behrens, J. a. Müller, J. Göke, K.M. Ritalahti, R. Wagner, E. Goltsman, A. Lapidus, S. Holmes, F.E. Löffler, and A.M. Spormann,“Localized plasticity in the streamlined genomes of vinyl chloride respiring *Dehalococcoides*.” *PLOS Genetics,* vol. 5, no. 11, pp. 1–10, 2009.

[6] M. Yohda, O. Yagi, A. Takechi et al.,“Genome sequence determination and metagenomic characterization of a *Dehalococcoides* mixed culture grown on *cis*-1,2-dichloroethene.” *Journal of Bioscience and Bioengineering,* vol. 120, no. 1, pp. 69–77, 2015.

[7] Y. Uchino, T. Miura, A. Hosoyama, S. Ohji, A. Yamazoe, M. Ito, Y. Takahata, K. Suzuki, and N. Fujita, “Complete genome sequencing of *Dehalococcoides* sp. strain UCH007 using a differential reads picking method.” *Standards in Genomic Sciences,* vol. 10, no. 102, pp. 1-7, 2015.

[8] R. Seshadri, L. Adrian, D .E. Fouts et al., “Genome sequence of the PCE-dechlorinating bacterium *Dehalococcoides ethenogenes,*” *Science* vol. 307, no. 5706, pp. 105–108, 2005.
